# Cerebrotendinous Xanthomatosis patients with late diagnosed in single orthopedic clinic: two novel variants in the *CYP27A1* gene

**DOI:** 10.1186/s13023-024-03082-4

**Published:** 2024-02-09

**Authors:** Muhammed Köroğlu, Mustafa Karakaplan, Enes Gündüz, Betül Kesriklioğlu, Emre Ergen, Okan Aslantürk, Zeynep Maraş Özdemir

**Affiliations:** 1 Orthopaedics and Traumatology Department, Turgut Özal Medical Center, İnönü University Medical School, Malatya, 44280 Turkey; 2 Orthopaedics and Traumatology Department, Şarkışla State Hospital, Sivas, Turkey; 3 Department of Medical Genetics, Cerrahpaşa Faculty of Medicine, İstanbul University, Istanbul, Turkey; 4 Department of Radiology, Turgut Özal Medical Center, İnönü University Medical School, Malatya, Turkey

**Keywords:** Cerebrotendinous Xanthomatosis, Novel mutation, Achilles, Tendon xanthomas

## Abstract

**Background:**

Cerebrotendinous Xanthomatosis (CTX) is a rare autosomal recessive lipid storage disorder caused by loss of function variants in the *CYP27A1* gene which encodes sterol 27-hydroxylase, on chromosome 2q35. Although the symptoms begin commonly in infancy, CTX diagnosis is often delayed. The aim of this study is to review the orthopedic findings of the disease by providing an overview of the clinical features of the disease. It is to raise awareness of this condition for which early diagnosis and treatment are important.

**Methods:**

We retrospectively evaluated the clinical, laboratory, radiological, and genetic findings of eight patients from four families who were admitted to our Orthopedics and Traumatology Department between 2017 and 2022 due to bilateral Achilles tendon xanthomas, were found to have high cholestanol and *CYP27A1* gene mutations.

**Results:**

The mean age of patients was 37, and five of them were male. The mean age at the onset of symptoms was 9.25 years. The mean age of initial diagnosis was 33.75 years. Between symptom onset and clinical diagnosis, an average delay of 24.5 years was observed. All patients had bilateral Achilles tendon xanthoma. Notably, a novel variant (c.670_671delAA) in CYP27A1 gene was identified in three patients who also presented with peripheral neuropathy and bilateral pes cavus. One patient had osteoporosis and four patients had osteopenia. Five patients had a history of bilateral cataracts. Furthermore, three of the patients had early-onset chronic diarrhea and three of the patients had ataxia. Two of the patients had epilepsy and seven of the patients had behavior-personality disorder. All patients had low intelligence, but none of them had cardiac disease.

**Conclusion:**

We present the diagnostic process and clinical features which the largest CTX case series ever reported from single orthopedic clinic. We suggest that patients with normal cholesterol levels presenting with xanthoma being genetically analyzed by testing at their serum cholestanol level, and that all siblings of patients diagnosed with CTX be examined.

**Supplementary Information:**

The online version contains supplementary material available at 10.1186/s13023-024-03082-4.

## Introduction

**Fig. 1:**
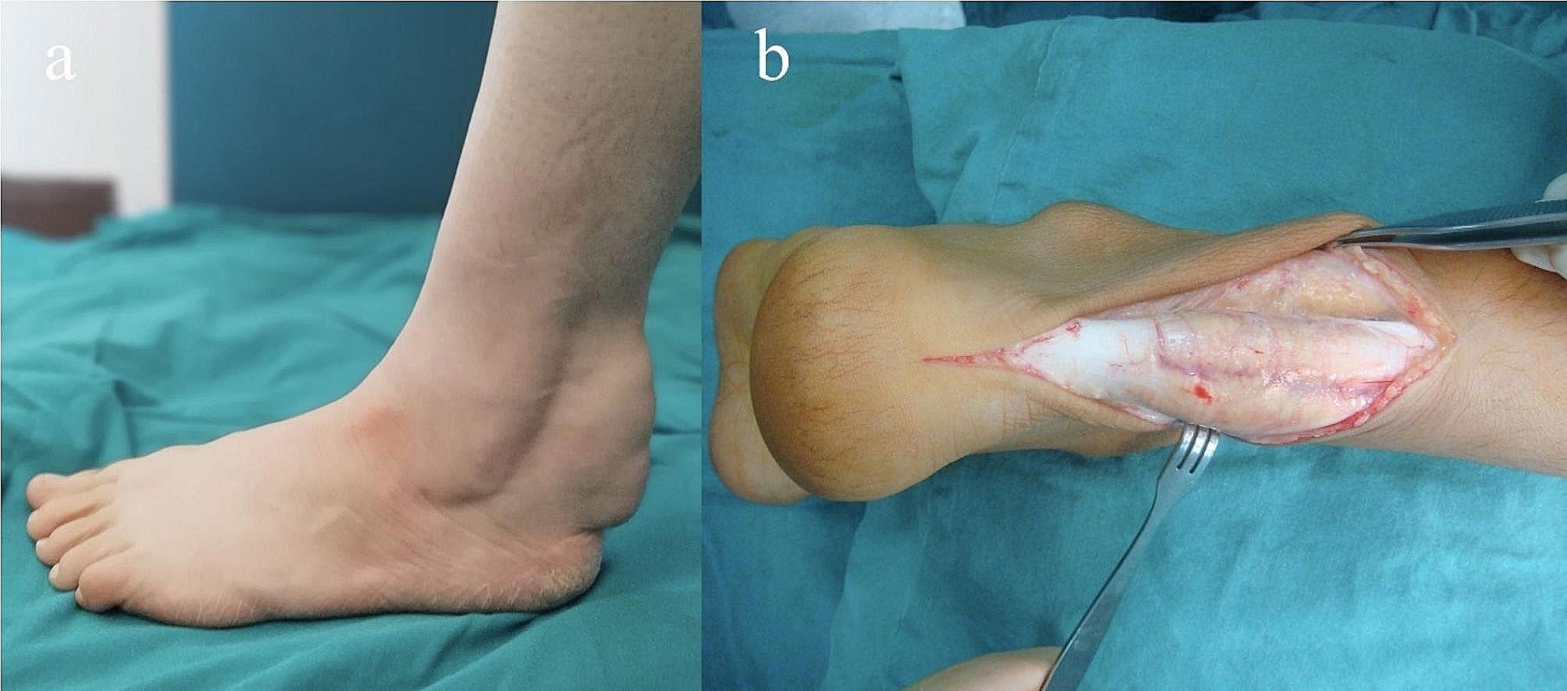
Left ankle, clinical photograph of the left ankle demonstrating the fusiform swelling caused by the xanthomata (patient 4) **(a)**, incision over the left Achilles tendon, note that the tendon was enlarged as a yellow firm hypertrophic neoplasm (patient 1) **(b)**

**Fig. 2:**
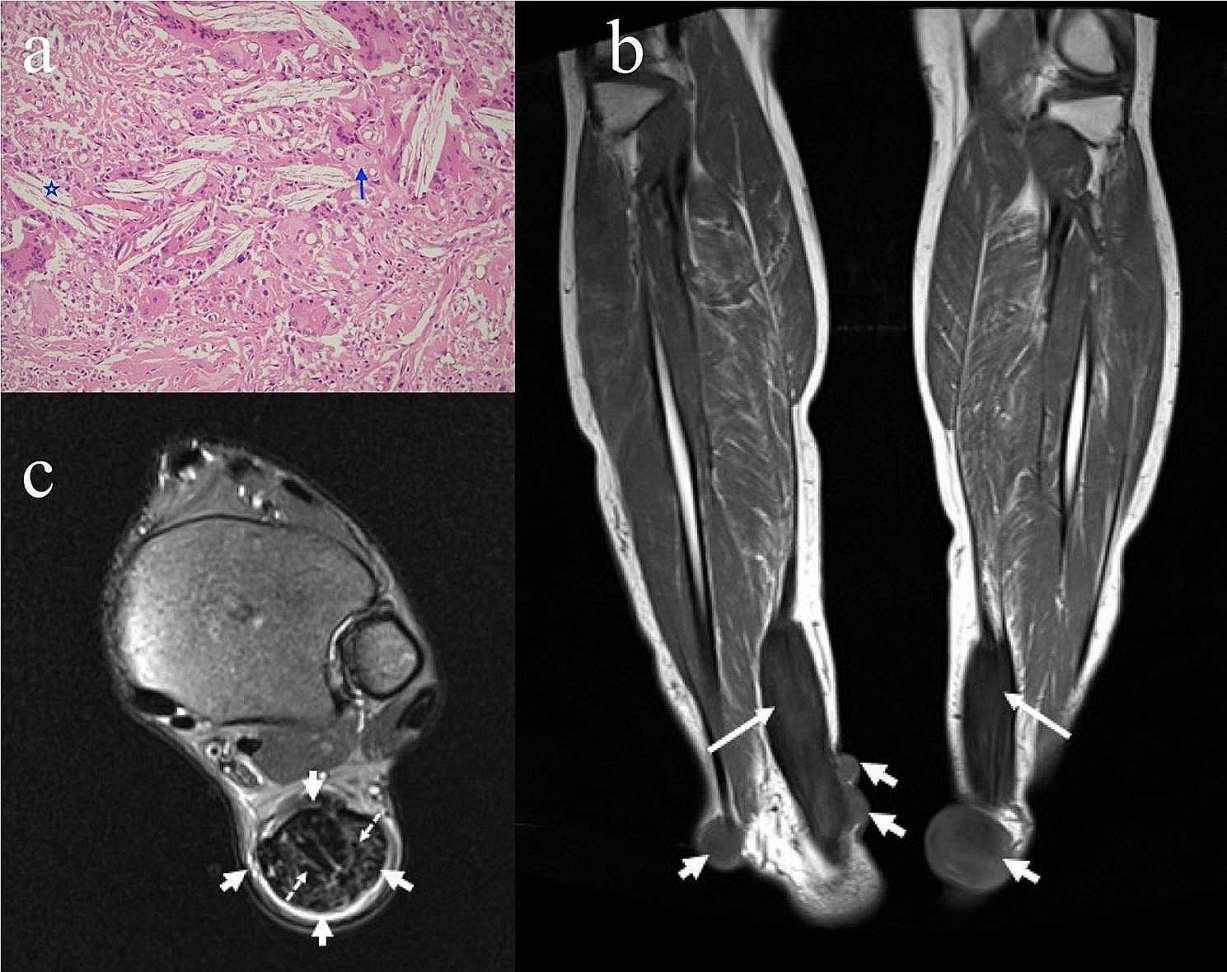
Photomicrograph of the specimen demonstrating cholesterol clefts (asterisk) and multinucleated giant cells (arrow) (Hematoxylin and eosin X200) (patient 1) **(a)**, coronal T1-weighted MR image of the lower leg, shows bilateral Achilles tendon xanthomas (long arrows) and multiple other tendon xanthomas around the bilateral ankle (short arrows) (patient 3) **(b)**, axial fat saturated T2-weighted MR image of the left ankle shows diffuse enlargement of the Achilles tendon (arrows) with characteristic speckled appearance (dotted arrows) (patient 4) **(c)**

In 1937, van Bogaert et al. published the first description of the uncommon condition of lipid metabolism known as Cerebrotendinous Xanthomatosis (CTX) [1]. Although there is no consensus on the exact prevalence of CTX, estimated rates are less than 5 per 100,000 people worldwide [2]. The prevalence of CTX may be higher than currently known. CTX (ORPHA:909/ OMIM: #213,700) is an autosomal recessive lipid storage disorder caused by pathogenic variants which leading to sterol 27-hydroxylase activity deficiency in the *CYP27A1* gene, on chromosome 2q35. Leitersdorf et al. demonstrated that the *CYP27A1* gene contains 9 exons and 8 introns and encompasses at least 18.6 kb of DNA [3]. Sterol 27-hydroxylase enzyme encoded by the *CYP27A1* gene is a member of the cytochrome P450 superfamily of enzymes. The mitochondrial enzyme catalyzes the first step in the oxidation of the side chain of sterol intermediates in the bile acid synthesis pathway [4]. This protein is important for overall cholesterol homeostasis, as the conversion of cholesterol to bile acids is the main pathway for the excretion of cholesterol from the body. Deficiency of the enzyme impairs the negative feedback of bile acids on 7α-hydroxylase, a rate-limiting enzyme in bile acid synthesis, and bile precursors are overproduced. As a result, bile acids cannot be synthesized and alternative pathways are activated leading to increased formation of cholestanol and 25-hydroxylated bile alcohols [5]. These patients characteristically have normal to low plasma low-density lipoprotein (LDL) and cholesterol levels in addition to their increased cholestanol levels [6]. Xanthomatous lesions consisting of lipid deposits have been shown in tendons, skin, lenses, brain, and spinal cord [7, 8]. Clinical features of this condition include tendon-skin xanthomatosis, early osteoporosis, cataracts, peripheral neuropathy, atherosclerosis, and progressive neurologic disorders (tremor, cerebral ataxia, intellectual disability, dementia, paresis, and psychiatric issues) [6, 8]. The initial signs of the disease may appear during the neonatal period as infantile-onset chronic diarrhea. Another remarkable first sign is juvenile cataract, which is usually diagnosed around the second decade [9]. Bilateral idiopathic cataract is common that in some cases it may has been the first manifestation of CTX [10, 11]. In neonate or infant whose get diarrhea and juvenile cataracts, these may be early symptoms of CTX [12]. Some neurologic symptoms usually start to appear late 20s or early 30s and in the absence of significant clinical signs such as xanthomas, CTX can be difficult to diagnose [6, 13]. Xanthomas generally manifest during the second or third decade (Fig. 1a and b) [13]. An enlarged tendon consists of giant multinuclear cells, fibroblasts, foam cells, and cholesterol crystal clusters (Fig. 2a) [14]. As well as the Achilles tendon, which is the main site of cholesterol and cholesterol esters deposits, tendon xanthomatosis can affect the triceps, tibial tuberosities, and fingers (Fig. 1b) [6]. Furthermore, tendon xanthomas are not a pathognomonic sign of CTX and can also develop in such as familial hypercholesterolemia, sitosterolemia, and occasionally primary biliary cholangitis [15]. There are typical similarities between the histologic characteristics of these xanthomas in CTX and hyperlipoproteinemia patients [6, 16]. However, plasma cholesterol levels are normal in CTX and distinguished from familial hypercholesterolemia where by normal cholesterol level [6, 9]. Another significant consideration in distinguishing CTX from familial hypercholesterolemia is the existence of a history of neurological involvement in CTX [17].

In addition to clinical findings, molecular analysis of the *CYP27A1* gene is utilized for definitive diagnosis of the disease [18]. Various types of mutations such as missense (most frequent), nonsense, splice site, small deletions, and insertions have been described before in all nine exons of *CYP27A1* [19]. Approximately half of the mutations in *CYP27A1* were detected in exons 6–8, followed by exon 2 and exon 4 [20, 21]. Most of these mutations affect adrenodoxin-binding (exon 6) or heme-binding (exons 8–9) sites critical for enzyme activity [18]. No genotype-phenotype correlations have been identified in CTX. Both inter- and intrafamilial phenotypic variability are observed [19, 22, 23].

In this study, we present the clinical characteristics of patients who applied to the orthopedics clinic because of Achilles tendon xanthomas and were diagnosed late. In this way, we aimed to describe the natural history of CTX by presenting the common early and late symptoms of the disease.

## Patients and methods

We retrospectively reviewed all clinical features, laboratory results, radiological imaging data, genetic analysis of eight CTX cases in four families at the Department of Orthopedics and Traumatology of our university over a six-year period from 2017 to 2022. All patients were positive for *CYP27A1* gene mutations and had increased serum cholestanol levels in this study. Patients who were not diagnosed by us and not followed up in our clinic were excluded from the study. We included the study adults over the age of 18, whom we followed for at least 6 months. After appropriate approval obtained the relevant authority at our institution for this retrospective study, informed consent was obtained from all participants.

We collected retrospectively data about the age, gender, consanguinity, follow-up time, height, weight, age at initial symptom, age at diagnosis, onset symptom, age at cataract surgery, existence and location of xanthomas with the age of onset, osteoporosis, skeletal deformity, diarrhea, epilepsy, ataxia, atherosclerosis, peripheral neuropathy, low intelligence, behavioral-personality disorder, and reduction in school performance of the patients.

In addition, information about the course and symptoms of the disease was obtained directly from patients, their first-degree relatives and caregivers. The data obtained from the *CYP27A1* gene mutation test, serum cholestanol, serum cholesterol, vitamin D, Ca, P, Electromyography (EMG), Dual-Energy X-ray Absorptiometry (DEXA), X-ray and Magnetic Resonance Imaging (MRI) examinations of the patients were evaluated. We determined the following spectrum of serum 25-Hydroxyvitamin D (25-OHD) levels: deficient if 25-OHD < 20 ng/mL, insufficient if 20 ng/mL < 25-OHD < 30 ng/mL, and normal if 30 ng/mL < 25-OHD < 100 ng/mL [24]. Clinical progression was also evaluated, with an average follow-up of four years.

**EMG.** The keypoint electroneurographic system (Dantec, Denmark) was selected to perform nerve conduction experiments, which were recorded using generally used standard methodologies. The motor and sensory nerve conduction velocities (m/s), the size of the amplitude compound motor action potentials (CMAP) (mV), the SNAP amplitude (V), and the distal motor and sensory latency (ms/cm) were all measured. All nerve conduction amplitudes and velocities were recorded orthodromically with reference values derived using previously established standards [25].

**DEXA.** Bone mineral density (BMD) measurements were performed in the anteroposterior (AP) view for the proximal hip DEXA scanner (Hologic QDR4500 Elite, Bedford, Mass). The BMD value for each region was calculated as the ratio of bone mineral content to the area of the interested region (g/cm2). For their T score values, the patients were grouped as having decreased bone mineral density (osteoporosis or osteopenia), and normal bone mineral density, according to the World Health Organization (WHO) diagnostic criteria for osteoporosis defined in 1994 [26].

**MRI**. All MRI examinations were performed on two 1.5- and 3-T scanners (Avanto and Skyra, Siemens Healthcare) using a dedicated coil. The participant was placed in the supine position on MRI examination table for both extremity and brain imaging. Our MRI protocol consisted of sagittal and axial T1-weighted and fat-saturated T2W or Short T1 Inversion Recovery (STIR), and coronal or axial T2W sequences for extremity (knee, lower leg, ankle) examination; axial T1W, T2W, fluid attenuation inversion recovery (FLAIR), and coronal and sagittal T2W sequences for brain examination. Magnetic resonance imaging assessments were performed by a radiologist with 15 years of MRI experience and 11 years of dedicated musculoskeletal imaging experience.

**Genetic analysis.** After obtaining informed consent, genetic tests of the patients were performed. Genomic DNA was isolated from the patient’s peripheral leukocytes using EZ1&2 DNA Blood Kit as described by the manufacturer (Qiagen, Germany). All exons and flanking regions of the *CYP27A1* gene were amplified by PCR using specific primers (specific primer sequences can be sent upon request). Nextera XT DNA Library Prep Kit (Illumina, San Diego, CA) was used for library preparation. The PCR-amplified target DNA region was sequenced on Illumina MiSeq platform (San Diego, CA, USA) using next generation sequencing. The variants were identified using the NCBI reference sequences NM_000784.4 / ENST00000258415 built on the GRCh38/hg38 reference genome. Pathogenicity and frequency data of variants was predicted using open-source programs such as Variant Effect Predictor (VEP)(https://www.ensembl.org/info/docs/tools/vep/index.html), Mutation Taster (https://www.mutationtaster.org/), dbSNP database (https://www.ncbi.nlm.nih.gov/SNP/), The Human Gene Mutation Database (HGMD) (http://www.hgmd.cf.ac.uk/ac/index.php), GnomAD (https://gnomad.broadinstitute.org/), Clinvar (https://www.ncbi.nlm.nih.gov/clinvar/), Franklin Genoox (https://franklin.genoox.com/clinical-db/home) and Varsome (https://varsome.com/). Detected variants were confirmed by Sanger sequencing.

### Patients

**Patient 1.** 33-year-old married female patient was 1.60 m tall and weighed 42 kg. She was born at term by normal spontaneous vaginal delivery and showed normal growth after birth. There was no consanguinity between her parents. Eighteen years ago, swelling developed in the Achilles tendon of both feet, and a large solid mass in each Achilles tendon, which grew slowly each year, was referred to our hospital six years ago because of ankle pain, bilateral pes cavus, and bilateral lower extremity numbness. We evaluated the patient’s four extremities using EMG. The patient had bilateral lower extremity peripheral neuropathy. Furthermore, DEXA revealed that the patient had osteoporosis. Following completion of the necessary examinations, we performed surgical excision of the Achilles xanthoma and transferred the flexor hallucis longus tendon (FHL) to the calcaneus. Pathological examination of the specimen revealed accumulation of cholesterol and cholesterol ester deposits within the excised mass (Fig. 1b). She reported irritability beginning in adolescence and continuing until the present. In addition, the patient had undergone bilateral cataract surgery for visual impairment at another hospital at the age of 12 years. On the other hand, she reported untreated infantile-onset chronic diarrhea. However, she did not have any speech impairment, tremor-ataxia, or gait disturbances. At the age of ten, she began to have difficulty understanding school lessons and dropped out of school after a period of declining educational performance. Anamnesis from his family revealed signs of changes in her behavior during primary school. After conducting the necessary investigations, we diagnosed the patient with CTX six years ago.

**Fig. 3:**
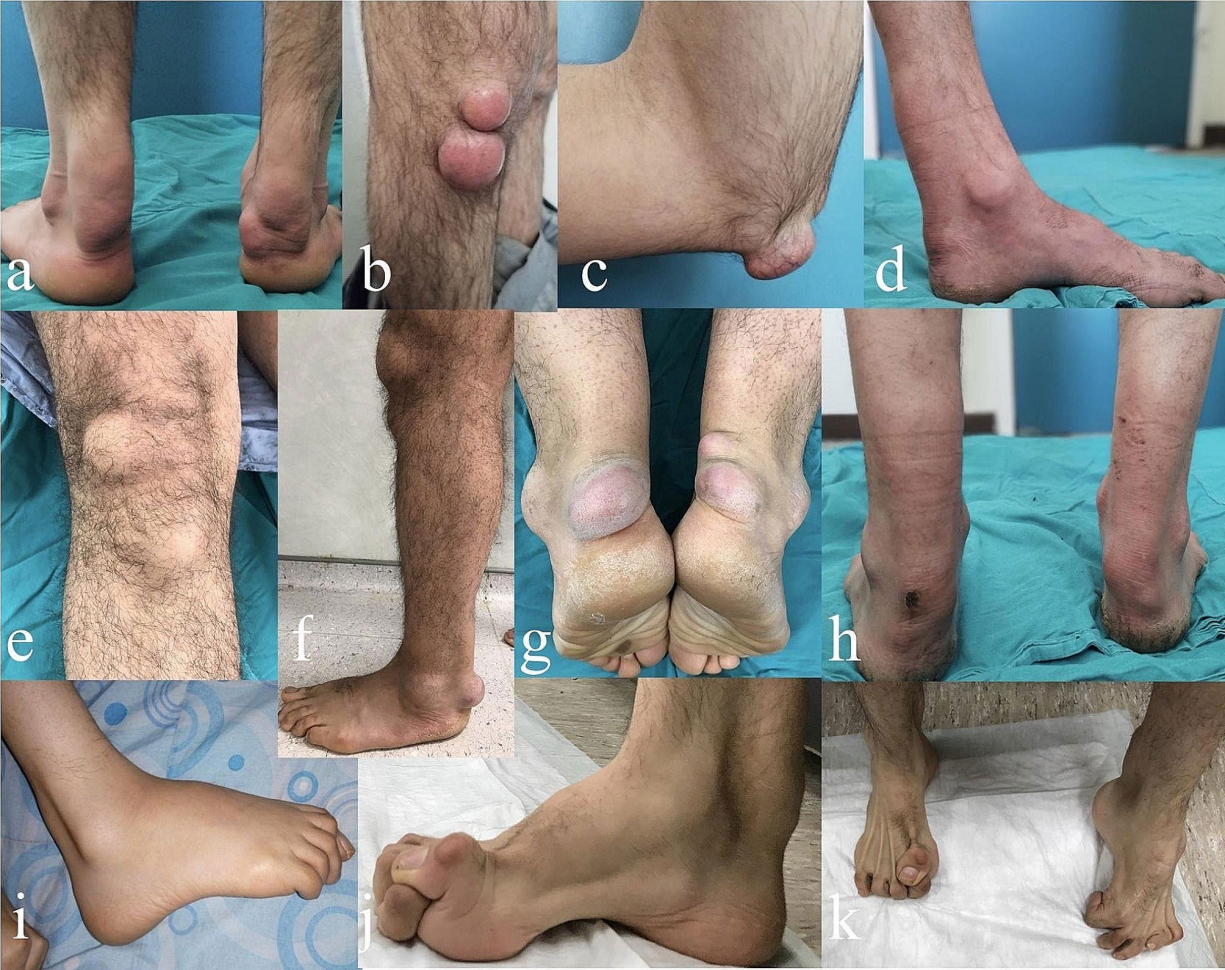
Clinical photographs of patient 2 showing bilateral ankle **(a)**, patellar tendon **(b)** and triceps tendon **(c)** xanthomas. Photographs of patient 5 that the left **(d)** and bilateral ankle **(h)** demonstrating the fusiform swelling caused by the xanthomas. Clinical photographs in the patient 3 that showing xanthoma in the right **(e)** and left **(f)** patellar tendon as well as multiple xanthomas in the bilateral Achilles tendons **(g)**. Photographs of patient 7 **(i)** and patient 6 showing Achilles tendons xanthomas **(j)** and foot deformities **(k)**

**Patient 2.** 28-year-old unmarried male patient was 1.70 m tall and weighed 65 kg. He was born via normal spontaneous vaginal delivery and did not experience any medical issues after birth. There was consanguinity between his parents. He was referred to us five years ago due to rapidly progressing swelling of the Achilles (Fig. 3a), patellar (Fig. 3b), and triceps tendons (Fig. 3c). The patient had no history of infantile-onset chronic diarrhea, vision-speech impairment, or cataract surgery. However, he reported experiencing tremors in both hands-ataxia symptoms for the past year and had difficulty understanding during his primary school years, which led to a decrease in school performance and leaving school at 11 years old. The patient’s family also reported changes in his personality beginning in early adolescence. The patient mentioned that his sister had experienced similar health issues. No peripheral sensory defects, gait disturbances, or foot deformities were reported. The patient was diagnosed with CTX five years ago.

**Patient 3.** 41-year-old male patient was 1.75 m tall and weighed 80 kg. He was married and had three children. He was born via normal spontaneous vaginal delivery and did not experience any medical issues after birth. There was consanguinity between his parents. Two years ago, the patient was referred to us owing to slowly progressive swelling of the bilateral Achilles (Fig. 3g) and patellar tendons (Fig. 3e-f). The patient had no history of infantile-onset chronic diarrhea, speech impairment, or tremor-ataxia symptoms. Nevertheless, he reported having undergone bilateral cataract surgery for visual impairment ten years previously. The patient stated that he had to leave school during primary school due to difficulties in comprehension and a decline in classroom performance at 13 years old. There were no reported changes in personality, peripheral sensory defects, gait disturbances, or foot deformities. After conducting the necessary investigations, we diagnosed the patient with CTX two years ago.

**Patient 4.** 30-year-old female patient was 1.72 m tall and weighed 59 kg. She was married and had three children. She was born at term by normal spontaneous vaginal delivery and showed normal growth after birth. There was consanguinity between her parents. The patient was referred to us five years ago due to rapidly progressing swelling of the Achilles tendon (Fig. 1a). The patient was diagnosed with osteopenia using DEXA. Moreover, the patient’s brother had similar health problems. The patient had no history of infantile-onset chronic diarrhea, vision-speech impairment, cataract surgery, or tremor-ataxia symptoms. Although, she reported difficulties with understanding during her primary school years, which led to deterioration in her school performance and her dropping out of school at 15 years old. The patient’s family also reported that her personality had begun to change during early adolescence. There were no reported peripheral sensory defects, gait disturbances, or foot deformities. CTX was diagnosed in the patient five years ago.

**Patient 5.** 44-year-old unmarried male patient was 1.69 m tall and weighed 55 kg. He was born via normal spontaneous vaginal delivery and did not experience any medical issues after birth. There was no consanguinity between his parents. Six years ago, he noticed swelling in the Achilles tendons (Fig. 3d and h), which slowly increased each year. The patient had undergone bilateral cataract surgery due to visual impairment at another hospital eight years ago. However, he did not report infantile-onset chronic diarrhea. Additionally, the patient stated that he had difficulty remembering what he had learned after 10 years. He did not have any speech disorders or ataxia symptoms but reported experiencing tremors in his bilateral hands that started in adolescence and continued to the present. At the age of nine, the patient began experiencing irritability and difficulty in understanding school lessons. Anamnesis from his family revealed signs of character changes during primary school. The patient did not complain of limb paresthesia, gait disturbance, or foot deformity during examination (Fig. 3d). The patient was diagnosed with osteopenia using DEXA. After conducting the necessary examinations, we diagnosed CTX three years ago.

**Patient 6.** 39-year-old male patient was 1.64 m tall and weighed 52 kg. He was born at term by normal spontaneous vaginal delivery and showed normal growth after birth. There was no consanguinity between his parents. He presented with a history of rapidly progressive swelling of both Achilles tendons that had first appeared ten years previously (Fig. 3j). Further investigation revealed a history of untreated infantile-onset chronic diarrhea, bilateral cataract surgery at the age of 12, and a diagnosis of epilepsy following seizures at the age of 13. Additionally, the patient reported difficulty in understanding during his primary school years, which led to a reduction in school performance and ultimately resulted in him leaving school at 13 years old. In adolescence, the patient began experiencing symptoms of irritability, tremor, speech impairment, ataxia, and personality changes. His family reported similar health issues in his siblings. The patient also suffered from peripheral sensory defects and gait disturbance over the previous five years, which progressively worsened to bedriddenness. We evaluated the patient’s four extremities using EMG. The patient had bilateral lower extremity peripheral neuropathy. He had bilateral pes cavus deformity (Fig. 3k). According to the DEXA scan, the patient had osteopenia. After completion of all necessary examinations, we diagnosed the patient with CTX five years ago.

**Patient 7.** 45-year-old unmarried male patient was 1.65 m tall and weighed 45 kg. He was born at term by normal spontaneous vaginal delivery. There was no consanguinity between his parents. He presented with a history of rapidly progressive swelling of both Achilles tendons, which had first appeared 15 years previously. He reported no other medical issues at birth or during the following years. Further investigation revealed a history of untreated infantile-onset chronic diarrhea, bilateral cataract surgery at the age of 12 years, and a diagnosis of epilepsy following seizures at the age of 13 years. In addition, the patient reported difficulties in learning during his primary school years, which led to a worsening of his educational performance and eventually to his school dropout at 13 years old. During adolescence, the patient experienced symptoms of irritability, speech problems, tremor, ataxia, and changes in his personality. His family also reported similar health issues in his siblings. Over the past years, the patient also experienced peripheral sensory defects and gait problems, which progressively worsened to the point where he was bedridden. We evaluated the patient’s four extremities with EMG. The patient had bilateral lower extremity peripheral neuropathy. He had bilateral pes cavus deformity (Fig. 3i). Based on the results of a DEXA scan, the patient was diagnosed with osteopenia. We diagnosed the patient with CTX five years ago.

**Patient 8.** 42-year-old unmarried female patient was 1.60 m tall and weighed 75 kg. She was born at term by normal spontaneous vaginal delivery and showed normal growth after birth. There was no consanguinity between her parents. The patient was referred to us one year ago due to slowly progressing swelling of the Achilles tendon which had occurred three years ago. Moreover, the patient’s brother had similar health problems. The patient had no history of infantile-onset chronic diarrhea, vision-speech impairment, cataract surgery, or tremor-ataxia symptoms. Although, she reported difficulties in understanding during her primary school years, which led to a deterioration in her school performance and ultimately resulted in her leaving school at 14 years old. The patient’s family also reported that her personality began to change in early adolescence. There were no reported peripheral sensory defects, gait disturbances, or foot deformities. CTX was diagnosed in the patient one year ago.

## Results

There were eight patients, five of them male, from four unrelated families. The average age of the patients was 37.3 years. The mean patient follow-up period was four years. Five patients had a family history of consanguineous marriages. The average age of diagnosis of CTX was 33.75 years. The mean age at the onset of symptoms was 9.25 years. According to the findings of this study, a delay of 24.5 years on average existed between the onset of symptoms and the clinical diagnosis. All patients were diagnosed with CTX following the appearance of xanthomas of the Achilles tendon. The mean age of development of Achilles tendon xanthoma was 30 years. The most common initial symptom was learning difficulties associated with early school leaving; the mean age of patients was 12.25 years. However, the most common late period symptoms were xanthomas of the Achilles tendon and behavioral-personality disorder.

The clinical results of eight patients with CTX were summarized in Table 1. The onset symptom was infantile diarrhea in three patients (**1,6,7**) at five, three, and four years of age respectively; five patients (**2,3,4,5,8**) had low intelligence associated with early school leaving in early adolescence. All patients were noted to have low intelligence over time. Interestingly, a novel mutation was found in three patients (**1,6,7**) whose initial symptom was infantile diarrhea. Five patients (**1,3,5,6,7**) had a history of bilateral cataracts. In particular, three of the cataract patients (**1,6,7**) had a history of juvenile bilateral cataracts and underwent surgery at 12 years, whereas the remaining two patients (**3,5**) with late onset cataracts underwent surgery at 31 and 36 years respectively, the mean age of cataract surgery was 20.6 years.

All patients had bilateral Achilles tendon xanthomas that developed at 15, 23, 39, 25, 38, 29, 30, and 41 years of age respectively. In addition to Achilles tendon xanthomas, two patients (**2,3**) also had bilateral patellar tendon xanthoma, and one patient (**2**) had bilateral triceps tendon xanthoma. Three patients **(2,6,7)** were being experienced the ataxia which began in adolescence. Two patients (**6,7**) were diagnosed with epilepsy, which began when they were 13 years old. Seven patients had behavior-personality disorder, but none had cardiac disease (Table 2).

All patients had increased serum cholestanol levels (between 5.77 and 35.27 µg/mL; normal < 3.75 µg/mL) during the diagnostic process. However, all patients had serum cholesterol levels within the normal range or upper limit (between 142 and 235 mg/dl; normal < 200 mg/dl). According to the laboratory analysis results, the patient’s serum calcium, and phosphate levels were normal. 25-OHD level was normal in two patients with osteoporosis (**1**) and osteopenia (**5**), insufficient in other three osteopenia patients (**4,6,7**), and deficient in three patients (**2,3,8**) with normal DEXA scores. In eight patients with CTX were assessed by analyzing their EMG recordings. The sensorimotor peripheral neuropathy was identified in three siblings (**1,6,7**) who had a novel gene mutation c.670_671delAA p.(Lys224ThrfsTer63). One of the patients (**1**) had early osteoporosis and four patients (**4,5,6,7**) had osteopenia as determined by DEXA. There was no history of pathological fracture in any of the patients.

**Fig. 4:**
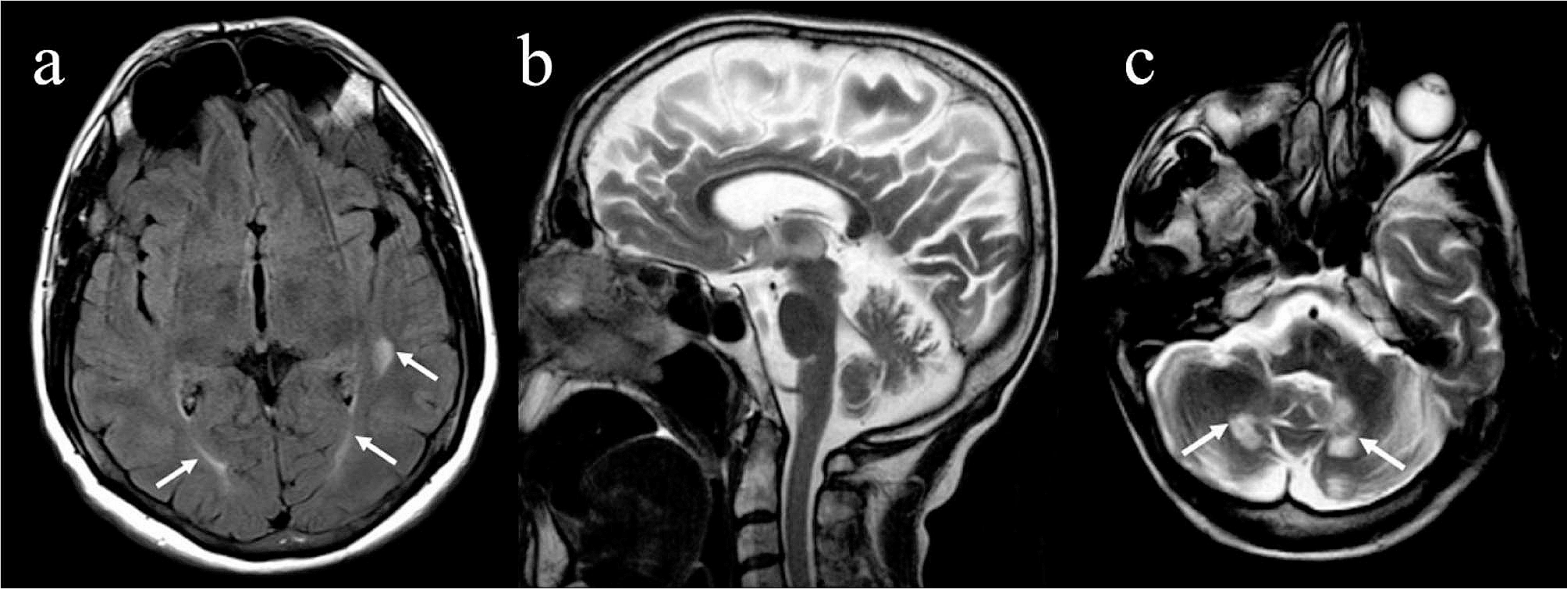
Axial FLAIR MR image (patient 7), at the level of thirth ventricule of the same patient revealed bilateral periventricular hyperintensity **(a)** (white arrows), sagittal **(b)** and axial **(c)** T2-weighted MR images show cerebral and cerebellar atrophy with prominence of the sulci and hyperintensity of the bilateral dentate nuclei (arrows)

The xanthomas that developed in the patients were evaluated by X-ray and MRI. Two patients (**2, 3**) had multiple tendon xanthomas around the ankle (Fig. 2b). Bilateral ankle MRI revealed that fusiform enlargement and loss of the normal anterior concavity of the Achilles tendon with the speckled appearance of the tendon in axial sequences (Fig. 2c). Brain MRI was performed in six patients (**1, 2, 3, 4, 6, 7**). The most frequent neuroimaging findings in the patients were non-specific periventricular white matter hyperintensities on T2W and FLAIR sequences (Fig. 4a). The other findings were cerebral and cerebellar atrophy in two patients (**6, 7**), hyperintensity of the bilateral caudate nucleus in a patient (**4**) and hyperintensity of the bilateral dentate nuclei and surrounding deep cerebellar white matter in three patients (**1, 6, 7**) (Fig. 4b, and Fig. 4c).

As a result of *CYP27A1* gene sequence analysis, homozygous missense variant c.409 C > T p.(Arg137Trp) was detected in four patients **(2,3,4,8)** and two heterozygous variants (c.409 C > T p.(Arg137Trp) and c.1263 + 4 A > T) were detected in one patient **(5)**. The c.670_671delAA p.(Lys224ThrfsTer63) homozygous frameshift variant was identified in three other patients who are siblings **(1,6,7)**. The c.409 C > T p.(Arg137Trp) variant has been previously described in disease-causing mutation databases such as the Human Gene Mutation Database (HGMD) Professional (CM940329) [27] and ClinVar. It was identified as a heterozygous variant in The Genome Aggregation Database (GnomAD, exomes), in 11 out of 251,112 analyzed alleles in healthy individuals (ƒ=0.00004381). The c.1263 + 4 A > T intronic variant was not reported in disease-causing mutation databases before. There is no GnomAD notification about the frequency. The variant has been classified as a “Variant of Uncertain Significance” according to American College of Medical Genetics / Association for Molecular Pathology (ACMG/AMP) criterias. CADD score is 24. The analysis of c.1263 + 4 A > T on the splicing process, using the Human Splicing Finder (http://www.umd.be/SSF/) program, predicted that most probably affecting splicing.

The c.670_671delAA p.(Lys224ThrfsTer63) variant was not reported in disease-causing mutation databases before. There is no GnomAD notification about the frequency. The variant has been classified as a “’Likely Pathogenic " according to American College of Medical Genetics / Association for Molecular Pathology (ACMG/AMP) criterias. A two-base deletion occurred between positions 670–671(exon 4) of the sequence encoding the protein. The frameshift mutation from position 224 of the protein caused a stop codon 63 codons later. Loss of function is a known disease mechanism in CTX. Null variants in same exon have been reported in ClinVar. The genetic and clinical evaluations of eight patients with CTX were summarized in Table 2.

## Discussion

The prevalence of CTX appears to be higher than is usually recognized and delayed diagnosis contributes to this situation [28]. It was found in the present study that the diagnosis delay was 24.5 years, which was higher than in previous studies [22, 23, 29–49]. The delayed interval in the diagnosis of CTX may be attributed, in part, to the diversity of neurological symptoms that characterize the condition, as well as the unspecific nature of early indications such as cataracts and diarrhea, lack of clinical experience in the rare diseases. Generally, the initial signs that prompt individuals to consult medical assessment are neurological anomalies, predominantly cognitive disorders. However, physicians should be vigilant to a history of chronic diarrhea and/or juvenile cataracts so that treatment can be initiated prior to the development of neurological manifestations [22]. In our study, the most observed initial neurological symptom was learning difficulties as an early symptom, while ataxia and peripheral neuropathy were observed as later neurological symptoms. Therefore, it is essential to exclude CTX as a differential diagnosis in cases of low intelligence. Mignarri et al. reviewed retrospectively 55 cases of CTX and found that intellectual disability and epilepsy were the earliest symptoms of the disease, while spasticity, ataxia, polyneuropathy, and parkinsonism were observed as late symptoms [29]. It is remarkable to mention that intellectual disability, which typically manifests during school age, warrants early consideration for diagnosis. In terms of systemic manifestations, the study identified tendon xanthomas and cataracts as the most frequent occurrences, with a prevalence rate of 69% and 88%, respectively. Furthermore, the onset of cataracts was usually observed in the second decade, whereas xanthomas were more commonly observed in the third decade [29].

In our study, compliance with the report by Mignarri et al., the most common initial symptom was learning difficulties associated with early school leaving, with an average age of 12.25. Otherwise, the most common late symptom was Achilles tendon xanthoma with a mean age of 30 years. In our study, although three patients were operated for cataract at the age of 12 years, the mean age of cataract surgery was 20.6 years. Verrips et al. published 14 CTX patients, there was no difference in age at onset, age at diagnosis, or delay in diagnosis between with and without xanthomas in patients. In this study stated that the existence of xanthomas is not imperative for the diagnosis of CTX [31]. However, in our study, all patients were presented with xanthomas of the Achilles tendon around the third decade of life. Only patient **(1)** stated that xanthoma severely interfered with her walking, the pain was exacerbated by walking or standing, limiting her walking distance. A surgical excision of Achilles xanthoma was performed, and Achilles reconstruction has been done with the FHL tendon. Four years later, we noticed that the Achilles xanthoma recurred and reached the same size again. Mondelli et al. reported that CDCA treatment slightly diminished the size of the xanthomas in some patients and stabilized the clinical severity in all cases, apparently preventing progression [32]. Conversely, the patient whom we treated surgically stated that his ankle complaints did not regress in two years of regular CDCA use before the operation. We did not detect a regression in the size of the existing xanthoma with CDCA treatment. We emphasize that patient compliance is significant in terms of drug use, surgery, and recurrence. Low intelligence and personality change are the two major factors that restrict patient compliance. We believe that the most important reason for low intelligence is the late diagnosis of patients. In CTX, osteoporotic patients generally have normal or decreased 25-OHD levels, normal plasma calcium and phosphate concentrations, and low bone mineral density [15]. The abnormal bile acid synthesis in CTX patients leads to marked alterations in calcium malabsorption, therefore resulting in an increase in bone resorption and causing osteoporosis. The bone mineral density increases following CDCA treatment had been shown in a study [33]. Martini et al. found a substantial rise in BMD after long-term CDCA therapy, particularly at the spine, also considering Z scores. In their study serum 25-OHD levels had been improved slightly but substantially after CDCA therapy: as none of their patients were taking calcium or vitamin D this may have been related to improved intestinal absorption of dietary vitamin D secondary to normalization of bile acid composition [34]. In our study, we detected osteoporosis in only one patient and osteopenia in four patients. Although these patients with normal Ca and P levels did not take any supplementary calcium or vitamin D, their 25-OHD levels were above 20 ng/mL. Berginer et al. reported that 14 CTX patients who were older than 26 years developed fractures [35]. However, none of our patients had a history of pathological fracture.

The etiology of peripheral neuropathy in CTX is still controversial in the literature. Some authors interpreted pathological process in nerves must have demyelinating in nature. Nevertheless, Verrips et al. suggested that distal axonopathy is the primary process in CTX [23]. They have demonstrated that myopathy is not a feature of CTX and that the most characteristic neuromuscular abnormality in ten patients’ biopsies study is sensorimotor axonal polyneuropathy [23]. Mondelli et al. reported in their study the electrophysiological follow-up of five CTX patients who underwent chronic therapy with CDCA for 11 years [32]. The authors observed that CDCA treatment improved all electrophysiological measures, despite the absence of clinical improvement in the early stages of the therapy. Conversely, untreated patients presented a decline in clinical and electrophysiological parameters. The effectiveness of the treatment on the neurological system has also been shown in children. van Heijst et al. reported that electrophysiologic measures improved in five children (three with 5-year follow-up) after treatment with CDCA. Neurological function and Intelligence Quotient (IQ) improved in three children, diarrhea ceased, and cataracts did not develop [36]. In our study, demyelinating motor neuropathy was identified by EMG in three siblings who were non-compliant with treatment, two of whom exhibited a significant decrease in mobility and was unable to ambulate independently around the fourth decade of life. Clinical findings of these patients (**1,6,7**) started with diarrhea and included juvenile cataract, osteopenia, bilateral pes cavus, and Achilles tendon xanthoma. In addition, a pathological examination of the operative specimen of one of these patients revealed the presence of cholesterol and cholesterol ester accumulation in the excised mass, which is a supportive and valuable finding for the diagnosis of CTX.

We detected a novel variant (c.670_671delAA p.(Lys224ThrfsTer63)) that was not previously described in the literature in these three siblings had peripheral neuropathy. In previous studies, deletion variants have been reported in CTX patients with predominant neurological symptoms [37–40].

Tao et al. reported peripheral neuropathy in 5 patients in their study including 6 patients in which they detected new mutations in 3 patients [40]. In their study, a novel probable pathogenic mutation (c.368_374delCCAGTAC,(p.Leu123fs)) and a previously reported pathogenic mutation (c.379 C > T, p.(Arg127Trp)) were found in a 32-year-old male patient who presented with gait disturbance for 20 years. At the age of 12 years, the patient developed progressive gait disturbance followed by leg spasms and paresis. On physical examination, short stature and bilateral pes cavus deformity were found. On neurological examination, bilateral muscle weakness, atrophy in the lower extremities, increased DTRs, and positive pathological reflexes were observed [40].

In our study c.1263 + 4 A > T and c.409 C > T variants were detected in patient **(5)**. Segregation analysis could not be performed because the family could not be reached. In addition to tendon xanthomas, the patient had late-onset cataract and cognitive disorders including behavioural abnormalities and low intelligence. Although splice site variants of the *CYP27A1* gene have been described previously, there are no publications related to the c.1263 + 4 A > T variant. Mutations affecting splicing in this region have been reported previously. For example, the c.1263 + 1 G > A variant has been previously reported as homozygous and compound heterozygous and has been demonstrated in cases of tendinous xanthoma with cataract, neurological and cognitive findings [30, 41–44].

The c.409 C > T variant has been reported in several previous publications as compound heterozygous or homozygous [45–48, 50]. Neurological involvement has been reported before in patients with this variant [45]. However, patients without neurological or cognitive symptoms have also been reported [46, 47]. Although ataxia was present in only one of our patients, behavioural disorders and low intelligence were present in 3 of 4 patients carrying this variant as homozygous. While cataract was not frequently observed in previous reports on this variant, late-onset cataract was observed in one of three members of the same family carrying this variant as homozygous in our study.

Cruysberg et al. published the first report of unexplained juvenile cataract and chronic infantile diarrhea as the earliest symptoms of pediatric CTX disease [51]. Cataract is thought to occur as a result of cholesterol-induced apoptosis in lens epithelial cells [9]. Verrips et al. reported a study that included 54 patients, it was found that bilateral premature cataract (90%) was the most common sign seen in CTX patients [50]. Bilateral juvenile cataract is important for early diagnosis and treatment because it appears before neurological symptoms and tendon xanthomas [51]. Yunisova et al. reported that the most common cardinal symptoms at onset were juvenile cataracts (85%) and unexplained chronic diarrhea (42%) [52]. Five (62.5%) of the eight patients in our study were operated for cataract, and only three (37.5%) of them had a history of bilateral juvenile cataracts. Bilateral juvenile cataract was not the initial symptom in any of these three patients. According to several reports, prolonged unexplained neonatal cholestasis may be the earliest sign of CTX [29, 49, 53]. However, Sekijima et al. did not report any patients with neonatal cholestasis, as in our study [49]. In terms of clinical findings, similar to the literature, both inter-family and intra-family phenotypic variability were observed in our study. No specific genotype-phenotype correlation was detected in CTX.

Early diagnosis of CTX holds major importance, as it enables the administration of disease-modifying therapies like CDCA, which can counteract metabolic disturbances and potentially ameliorate the neurological impairment linked with this disorder. Notably, the efficacy of treatment may decrease once neurological symptoms have fully manifested, possibly due to the irreversibility of lesions. Early initiation of therapy is believed to be more effective in mitigating the disease progression [49]. Throughout the study, we observed suboptimal adherence to treatment among all patients and noticed a correlation between noncompliance and progressive disease outcomes without statistical analysis. In conclusion, by the fourth decade of life, two patients exhibited a significant decline in mobility and could no longer ambulate independently.

## Limitations

The identified novel variants were evaluated in combination with predictions from *in silico* databases and clinical, laboratory, radiological, and pathological findings of patients. The pathogenicity of the novel variants should be confirmed by functional and biochemical studies. This is the limitation of our study and will be our next aim.

In this study, we discuss the phenotypic presentation of two novel variants. When we compare our patients with the previously described variant with the cases reported in the literature, we observe phenotypic variability; other possible causes of these variable findings may need to be investigated.

## Conclusions

This is the first report of the largest CTX patient group ever reported in the literature from a single orthopedic clinic. Eight patients were diagnosed after detailed examinations of patients who came to our clinic caused by xanthoma of the Achilles tendon. We emphasize that patients who are not diagnosed in a timely may present to an orthopedic surgeon with fusiform swelling caused by the xanthomata of the Achilles tendon complaints after a long period of time. Our goal in the study is to create the required awareness to achieve the primary diagnosis. We suggest that the occurrence of any of the four clinical features of CTX (progressive neurologic symptoms, premature cataracts, intractable diarrhea, and tendon xanthomas) with a positive family history should lead to a comprehensive metabolic evaluation for CTX. Affected relatives may be asymptomatic. Therefore, we recommend that all patients presenting with xanthoma being genetically analyzed by testing at their serum cholestanol level, and that all siblings of patients diagnosed with CTX be examined.

**Table 1:** Patients’ clinical data and laboratory results

Age and Sex (Case)	CM	AOS (Years)	Age of Diagnosis (Years)	Follow-up Time (Years)	Weight-Size (kg/m)	Cholestanol (µg/mL)	Cholesterol (mg/dl)	Vitamin-D (ng/mL)	Ca/P (mg/dl)	EMG (Peripheral Neuropathy)	DEXA (Total Hip Region)
**33 F (1)**		5 Diarrhea	27	6	42/160	35.27	186	35.94	9.5/3.7	+	BMD:0.536 T (-3.3) Z (-3.3)
**28 M (2)**	+	11 Low Intelligence	23	5	65/170	11.82	230	13.44	10.2/2.8		BMD:0.996 T (-0.2) Z (-0.2)
**41 M (3)**	+	13 Low Intelligence	39	2	80/175	7.58	211	17.6	9.5/2		BMD:1.089 T (0.4) Z (0.6)
**30 F (4)**	+	15 Low Intelligence	25	5	59/172	5.77	196	37.56	10.1/3.8		BMD:0.814 T (-1.1) Z (-1)
**44 M (5)**	+	9 Low Intelligence	41	3	55/169	18.87	235	39.61	9.6/2		BMD:0.746 T (-1.6) Z (-1.3)
**39 M (6)**		3 Diarrhea	34	5	52/164	29.41	186	22.45	9.2/3.3	+	BMD:0.631 T (-1.7) Z (-1.6)
**45 M (7)**		4 Diarrhea	40	5	45/165	11.01	142	20.67	8.5/2.4	+	BMD:0.780 T (-1.4) Z (-1.5)
**42 F (8)**	+	14 Low Intelligence	41	1	75/161	15.14	174	16.01	9.2/3.4		BMD:0.885 T (-0.5) Z (-0.3)

F Female; M Male; CM consanguineous marriages; AOS Age of onset and symptom; BMD Bone mineral density

**Table 2:** The genetic and clinical evaluations of eight patients

(Patients) CYP27A1 gene analysis	Cataract Age of surgery	Infantile Diarrhea	Tendon Xanthomas Onset	Osteoporosis	Pes Cavus	Ataxia	Behavioral personality disorder	Low Intelligence	Epilepsy	Atherosclerosis
**(1)** c.670_671delAA p.(Lys224ThrfsTer63)	Bilateral (Juvenile) (12y)	**+**	Bilateral Achilles Tendon (15y)	**+**	**+** Bilateral	**-**	**+**	**+**		
**(2)** c.409 C > T p.(Arg137Trp)			Bilateral Achilles+Patellar Tendon+Triceps Tendon (23y)			**+**	**+**	**+**		
**(3)** c.409 C > T p.(Arg137Trp)	Bilateral (Late onset) (31y)		Bilateral Achilles+ Bilateral Patellar Tendon (39y)					**+**		
**(4)** c.409 C > T p.(Arg137Trp)			Bilateral Achilles Tendon (25y)	Osteopenia			**+**	**+**		
**(5)** c.1263 + 4 A > T with c.409 C > T p.(Arg137Trp)	Bilateral (Late onset) (36y)		Bilateral Achilles Tendon (38y)	Osteopenia			**+**	**+**		
**(6)** c.670_671delAA p.(Lys224ThrfsTer63)	Bilateral (Juvenile) (12y)	**+**	Bilateral Achilles Tendon (29y)	Osteopenia	+ Bilateral	**+**	**+**	**+**	**+**	
**(7)** c.670_671delAA p.(Lys224ThrfsTer63)	Bilateral (Juvenile) (12y)	**+**	Bilateral Achilles Tendon (30y)	Osteopenia	+ Bilateral	**+**	**+**	**+**	**+**	
**(8)** c.409 C > T p.(Arg137Trp)			Bilateral Achilles Tendon (41y)				**+**	**+**		

### Electronic supplementary material

Below is the link to the electronic supplementary material.

Supplementary Material 1

## Data Availability

All data generated or analyzed during this study are included in this published article.

## Abbreviations

CTX: Cerebrotendinous Xanthomatosis
CDCA: chenodeoxycholicacid
LDL: low-density lipoprotein
EMG: Electromyography
DEXA: Dual-Energy X-ray Absorptiometry
MRI: Magnetic Resonance Imaging
25-OHD: 25-Hydroxyvitamin D
CMAP: Compound motor action potentials
BMD: Bone mineral density
WHO: World Health Organization
STIR: Short T1 Inversion Recovery
FLAIR: Fluid attenuation inversion recovery
FHL: Flexor hallucis longus tendon
HGMD: Human Gene Mutation Database
ACMG: American College of Medical Genetics
HSF: Human Splicing Finder
